# A 4-trifluoromethyl analogue of celecoxib inhibits arthritis by suppressing innate immune cell activation

**DOI:** 10.1186/ar3683

**Published:** 2012-01-17

**Authors:** Asako Chiba, Miho Mizuno, Chiharu Tomi, Ryohsuke Tajima, Iraide Alloza, Alessandra di Penta, Takashi Yamamura, Koen Vandenbroeck, Sachiko Miyake

**Affiliations:** 1 Department of Immunology, National Institute of Neuroscience, National Center of Neurology and Psychiatry, 4-1-1 Ogawahigashi, Kodaira, Tokyo 187-8502, Japan; 2 Neurogenomiks Laboratory, Universidad Del País Vasco (UPV/EHU), Parque Tecnológico de Bizkaia, 48170 Zamudio, Spain; 3 IKERBASQUE, Basque Foundation for Science, 48011, Bilbao, Spain

## Abstract

**Introduction:**

Celecoxib, a highly specific cyclooxygenase-2 (COX-2) inhibitor has been reported to have COX-2-independent immunomodulatory effects. However, celecoxib itself has only mild suppressive effects on arthritis. Recently, we reported that a 4-trifluoromethyl analogue of celecoxib (TFM-C) with 205-fold lower COX-2-inhibitory activity inhibits secretion of IL-12 family cytokines through a COX-2-independent mechanism that involves Ca^2+^-mediated intracellular retention of the IL-12 polypeptide chains. In this study, we explored the capacity of TFM-C as a new therapeutic agent for arthritis.

**Methods:**

To induce collagen-induced arthritis (CIA), DBA1/J mice were immunized with bovine type II collagen (CII) in Freund's adjuvant. Collagen antibody-induced arthritis (CAIA) was induced in C57BL/6 mice by injecting anti-CII antibodies. Mice received 10 μg/g of TFM-C or celecoxib every other day. The effects of TFM-C on clinical and histopathological severities were assessed. The serum levels of CII-specific antibodies were measured by ELISA. The effects of TFM-C on mast cell activation, cytokine producing capacity by macophages, and neutrophil recruitment were also evaluated.

**Results:**

TFM-C inhibited the severity of CIA and CAIA more strongly than celecoxib. TFM-C treatments had little effect on CII-specific antibody levels in serum. TFM-C suppressed the activation of mast cells in arthritic joints. TFM-C also suppressed the production of inflammatory cytokines by macrophages and leukocyte influx in thioglycollate-induced peritonitis.

**Conclusion:**

These results indicate that TFM-C may serve as an effective new disease-modifying drug for treatment of arthritis, such as rheumatoid arthritis.

## Introduction

In the past decade, a series of potent new biologic therapeutics have demonstrated remarkable clinical efficacy in several autoimmune diseases, including rheumatoid arthritis (RA). In the case of RA, a chronic progressive autoimmune disease that targets joints and occurs in approximately 0.5 to 1% of adults, biologic agents, such as TNF inhibitors, have proven effective in patients not responding to disease-modifying anti-rheumatic drugs, such as methotrexate. However, about 30% of patients treated with a TNF inhibitor are primary non-responders. Moreover, a substantial proportion of patients experience a loss of efficacy after a primary response to a TNF inhibitor (secondary non-responders) [1-3]. More recently, as new therapies have become available, including biological agents targeting IL-6, B cells and T cells, it has become clear that a notable proportion of patients respond to these new biological agents even among primary and secondary non-responders to TNF inhibitors [3-10]. These individual differences in response to each agent highlight the difficulty and limit of treating multifactorial disease by targeting single cytokine or single cell type. Patient-tailored therapy might be able to overcome this issue, but good biomarkers to predict treatment responses have not yet been elucidated.

Therefore, as described above, biological drugs have limited values. In addition, such drugs may be accompanied by serious side effects [11,12]. Furthermore, the high cost of these biological drugs may make access to these reagents prohibitive for the general public. Alternative therapeutic options, such as small molecule-based drugs, continue to be an important challenge.

The involvement of prostaglandin pathways in the pathogenesis of arthritis has been shown in animal models by using mice lacking genes, such as cycolooxygenase-2 (COX-2), prostaglandin E synthase, or prostacyclin receptor [13-15]. As COX-2 knockout mice normally develop autoreactive T cells in collagen-induced arthritis (CIA) [13], prostaglandin pathways appear to be involved mainly in the effector phase of arthritis. However, treatment with celecoxib, a prototype drug belonging to a new generation of highly specific COX-2 inhibitors has been reported to have only mild suppressive effects on animal models of arthritis, and strong inhibition of arthritis was achieved only when mice were treated in the combination of celecoxib with leukotriene inhibitors [16-19]. In humans, although celecoxib is widely used as an analgesic agent in patients with RA or osteoarthritis, there is no evidence that celecoxib therapy modulates the clinical course of RA. In addition, recently it has been shown that celecoxib enhances TNFα production by RA synovial membrane cultures and human monocytes [20].

Celecoxib has been reported to exhibit COX-2-independent effects, such as tumor growth inhibition and immunomodulation [21,22]. Previously, we demonstrated that celecoxib treatment suppressed experimental autoimmune encephalomyelitis (EAE) in a COX-2 independent manner [22]. We recently developed a trifluoromethyl analogue of celecoxib (TFM-C; full name: 4-[5-(4-trifluoromethylphenyl)-3-(trifluoromet-hyl)-1*H*-pyrazol-1-yl]benzenesulfonamide), with 205-fold lower COX-2-inhibitory activity. In studies using recombinant cell lines, TFM-C inhibited secretion of the IL-12 family cytokines, IL-12, p80 and IL-23, through a COX-2-independent, Ca^2+^-dependent mechanism involving chaperone-mediated cytokine retention in the endoplasmic reticulum coupled to degradation via the ER stress protein HERP [23,24]. In the present study, we demonstrate that TFM-C inhibits innate immune cells and animal models of arthritis, including CIA and type II collagen antibody-induced arthritis (CAIA), in contrast to the limited inhibitory effect of celecoxib. TFM-C suppresses the activation of mast cells in arthritic joints. Moreover, TFM-C treatment suppresses the production of inflammatory cytokines by macrophages and leukocyte recruitment. These findings indicate that TFM-C may serve as an effective new drug for the treatment of arthritis, including RA.

## Materials and methods

### Differentiation and stimulation of U937 cells

Human U937 cells were obtained from the American Type Culture Collection (Rockville, MD, USA) and cultured in RPMI 1640 supplemented with 10% FCS. To differentiate U937 cells, 5 × 10^5 ^cells were treated with PMA (25 ng/ml) for 24 hours. At 22 hours of PMA treatment, 50 μM of TFM-C was added for 2 hours. Subsequently, cells were stimulated with 5 μg/ml of LPS and PMA (25 ng/ml) for 0, 3, 6, 12 and 24 hours in the presence or absence of TFM-C. Supernatants were harvested and assayed for cytokine production by means of Quansys Q-Plex™ Array (Quansys Bioscience, Logan, Utah, USA). RNA isolation was performed following the manufacturer's instructions (Macherey-Nagel, Düren, Germany).

### Quantitative RT-PCR (qPCR)

A total of 200 ng of RNA extracted from U937 cells was retrotranscribed to cDNA using random primers according to the manufacturer's protocol (Applied Biosystems, Carlsbad, California, USA). qPCR was performed with the Supermix for SsoFast EvaGreen (Biorad, Hercules, California, USA) on a 7500 Fast Real-Time PCR System (Applied Biosystems). For each target gene, qPCR QuantiTect Primer Assays were used (Qiagen Hilden, Germany). For each sample, expression levels of the transcripts of interest were compared to that of endogenous GAPDH. The levels of mRNA are calculated as 2^-Ct^.

### Quansys Q-Plex™ Array chemiluminescent

A total of 30 μl of medium from differentiated U937 cells treated with PMA/LPS/TFM-C or LPS/PMA were analyzed. Human Cytokine Stripwells (16-plex) were used following the manufacturer's instructions. The image was acquired using Bio-Rad Chemidoc camera and analyzed with Q-View Software (Quansys Bioscience, Logan, Utah, USA)

### DAPI staining

Differentiated U937s were treated with LPS/PMA/TFM-C for 6, 12 and 24 hours and then fixed with 2% PFA. The cells were washed three times with PBS and then incubated with DAPI (1:50000; Molecular Probes, Carlsbad, California, USA) in PBS. Coverslips were embedded in Fluoro-Gel (Electron Microscopy Science, Hatfield, Pennsylvania, USA). Images were recorded using the ApoTome system (AxioVision, Carl Zeiss, Inc., Oberkochen, Germany) and analyzed using the ImageJ program (version 1.40, Bethesda, Maryland, USA).

### AlarmBlue staining of U937 cells

The number of viable cells was tested at 6, 12, and 24 hours after TFM-C exposure by adding the AlamarBlue reagent (AbD Serotec, Cambridge, UK). Absorbance was measured at wavelengths of 570 nm and 600 nm after required incubation, using a Varioskan Flash (Thermo Fisher Scientific, Fremont, CA, USA). Absorbance values of samples were normalized with values of the cell culture media without cells. The results are presented as the proportion of viable cells, calculated by dividing the absorbance values of drug-treated samples by the absorbance values of untreated control samples.

### Mice

DBA1/J mice were purchased from Oriental Yeast Co., Ltd. (Tokyo, Japan). C57BL/6J (B6) mice were purchased from CLEA Laboratory Animal Corp. (Tokyo, Japan). Animal care and use were in accordance with institutional guidelines and all animal experiments were approved by the Institutional Animal Care and Use Committee of the National Institute of Neuroscience.

### Induction of CIA

DBA1/J male mice (n = 5 to 6 per group, 7 to 8 weeks old) were immunized intradermally at the base of the tail with 150 μg of bovine type II collagen (CII) (Collagen Research Center, Tokyo, Japan) emulsified with an equal volume of complete Freund's adjuvant (CFA), containing 250 μg of H37Ra *Mycobacterium tuberculosis (Mtb) *(Difco, Detroit, MI, USA). DBA1/J mice were boosted 21 days after immunization by intradermal injection with 150 μg of CII emulsified with incomplete Freund's adjuvant (IFA).

### Induction of CAIA

B6 female mice (n = 5 to 6 per group, 7 to 8 weeks old) were injected intravenously with 2 mg of a mixture of anti-CII monoclonal antibodies (mAbs) (Arthrogen-CIA mAb (Chondrex. LLC. Seattle, WA, USA)), and two days later with 50 μg of lipopolysaccharide (LPS) was injected intraperitoneally.

### Clinical assessment of arthritis

Mice were examined for signs of joint inflammation and scored as follows: 0: no change, 1: significant swelling and redness of one digit, 2: mild swelling and erythema of the limb or swelling of more than two digits, 3: marked swelling and erythema of the limb, 4: maximal swelling and redness of the limb and later, ankylosis. The average macroscopic score was expressed as a cumulative value for all paws, with a maximum possible score of 16.

### Thioglycollate-induced peritonitis

Mice were injected with 1 ml of 4% sterile thioglycollate intraperitoneally. Four hours later, mice were killed and peritoneal lavage fluid was collected by washing the peritoneal cavity with cold PBS containing 5 mM EDTA and 10 U/ml heparin.

### Administration of TFM-C or celecoxib

TFM-C and celecoxib were synthesized as previously described [23]. We injected TFM-C or celecoxib intraperitonealy (i.p.) in 0.5% Tween/5% DMSO/PBS. In CIA experiments, mice received 10 μg/g TFM-C or celecoxib every other day from 21 days after immunuization. In CAIA, we injected the mice with 10 μg/g of TFM-C or celecoxib every other day starting at two days before disease induction. In thioglycollate-induced peritonitis experiments, mice received 10 μg/g of TFM-C or celecoxib two days and one hour before thioglycollate injection. The control animals were injected with vehicle alone.

### Histopathology

Arthritic mice were sacrificed and all four paws were fixed in buffered formalin, decalcified, embedded in paraffin, sectioned, and then stained with H&E. Histological assessment of joint inflammation was scored as follows: 0: normal joint, 1: mild arthritis: minimal synovitis without cartilage/bone erosions, 2: moderate arthritis: synovitis and erosions but joint architecture maintained, 3: severe arthritis; synovitis, erosions, and loss of joint integrity. The average of the macroscopic score was expressed as a cumulative value of all paws, with a maximum possible score of 12.

Mast cells in synovium were visually assessed for intact versus degranulating mast cells using morphologic criteria. Mast cells were identified as those cells that contained toluidine blue-positive granules. Only cells in which a nucleus was present were counted. Degranulating cells were defined by the presence of granules outside the cell border with coincident vacant granule space within the cell border as described previously [25].

### Measurement of CII specific IgG1 and IgG2a

Bovine CII (1 mg/ml) was coated onto ELISA plates (Sumitomo Bakelite, Co., Ltd, Tokyo, Japan) at 4°C overnight. After blocking with 1% bovine serum albumin in PBS, serially diluted serum samples were added onto CII-coated wells. For detection of anti-CII Abs, the plates were incubated with biotin-labeled anti-IgG1 and anti-IgG2a (Southern Biotechnology Associates, Inc., Brimingham, AL, USA) or anti-IgG Ab (CN/Cappel, Aurora, OH, USA) for one hour and then incubated with streptavidin-peroxidase. After adding a substrate, the reaction was evaluated as OD_450 _values.

### Stimulation of or macrophages

B6 mice received 10 μg/g of TFM-C or control vehicle on Day 0 and Day 2, and on Day 3, splenic macrophages were collected and were stimulated by LPS *in vitro *in the presence of TFM-C or vehicle.

### Detection of cytokines

Cytokine levels in the culture supernatant were determined by using a sandwich ELISA. The Abs for IL-1β ELISA were purchased from BD Biosciences (San Jose, CA, USA) and the ELISA Abs for IL-6 and TNFα were purchased from eBioscience (San Diego, CA, USA).

### Statistical analysis

CIA and CAIA clinical or pathological scores for groups of mice are presented as the mean group clinical score + SEM, and statistical differences were analyzed with a non-parametric Mann-Whitney *U*-test. Data for cytokines were analyzed by an unpaired t-test.

## Results

### TFM-C inhibits cytokine secretion from activated U937 cells concomitant with induction of an ER stress response {2^nd ^Level Heading}

**Figure 1:**
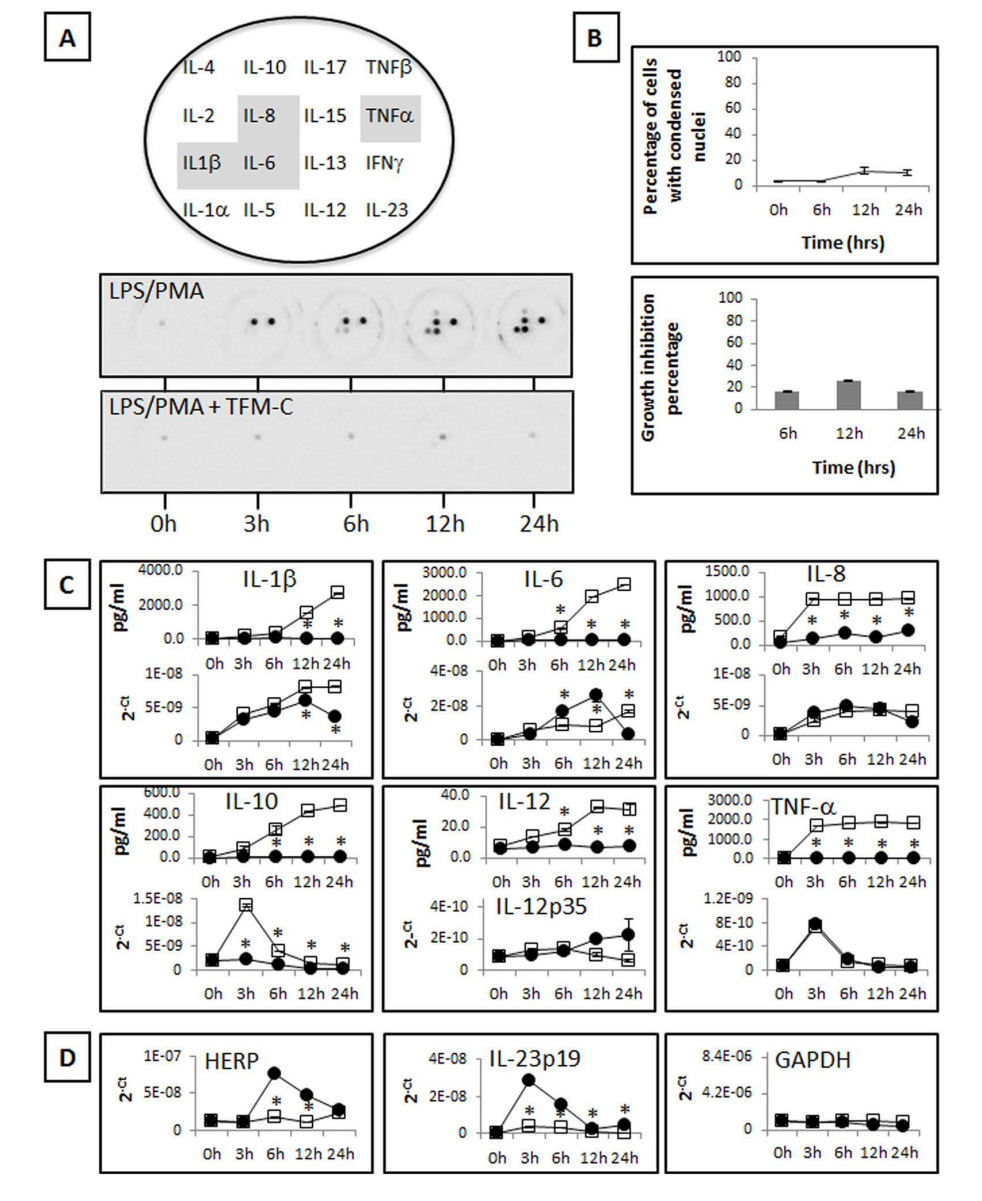
**Effect of TFM-C on cytokine production from activated U937 macrophages. A**. Lay-out of cytokine-specific antibody spots in the 16-plex cytokine Stripwell array (upper image) and visualization of cytokine-specific chemiluminescence in culture medium of LPS/PMA-treated U937 cells in the absence or presence of TFM-C (lower images). The grey-shaded cytokines in the upper images are those showing the highest production in LPS/PMA-treated U937 cells at 24 h. **B**. Effect of TFM-C treatment (50 μM) on the viability of macrophages (PMA-stimulated U937 cells). Apoptotic cells were measured by DAPI staining, and the percentage of damaged DNA and condensed chromatin was calculated following 6, 12 and 24 h of TFM-C treatment (upper graph). Metabolic activity of cells, measured by AlarmBlue^®^, was expressed as growth inhibition percentage of untreated controls for 6, 12 and 24 h of TFM-C treatment (lower graph). Bars show average of three independent experiments with corresponding error bars. **C**. Quantification of the kinetics of cytokine secretion and mRNA production (IL-1β, IL-6, IL-8, IL-10 IL-12 and TNF-α) in differentiated macrophages treated with LPS/PMA in the absence (open squares) or presence (solid circles) of TFM-C. All values represent the averages of three independent experiments. For each cytokine, the upper graph represents amount of secreted cytokine quantified using Quansys 16-plex Stripwells, while the lower graph represents cytokine-specific mRNA quantified by QPCR. Asterisks indicate significant differences at * *P *< 0.05 between TFM-C-treated and -untreated cells at each time point using Student's *t*-test. **D**. Effect of 50 μM TFM-C on IL-23p19, HERP and GAPDH mRNAs (QPCR) in differentiated macrophages, stimulated by LPS and PMA. The levels of mRNA levels are shown as 2^-Ct^. Asterisks indicate significant differences at * *P *< 0.05 compared with baseline condition LPS/PMA-only using Student's *t*-test.

In a recombinant cell system, TFM-C inhibits IL-12 secretion via a mechanism involving the induction of ER stress coupled to intracellular degradation of the cytokine polypeptide chains via the ER stress protein HERP [23,24,26]. In order to verify whether the cytokine secretion-inhibitory effect of TFM-C extends to natural cytokine producer cells, we assessed its effect using PMA/LPS-activated U937 macrophages, a well-known source of multiple cytokines. TFM-C potently blocked secretion of IL-β, IL-6, IL-8, IL-10, IL-12 and TNF-α (Figure 1A, C). By means of QPCR, TFM-C was found to suppress mRNA production of IL-10 over the course of the experiment, and at 12 and 24 h of TFM-C treatment, of IL-1β. Virtually no effect was seen on mRNA production of TNF-α and IL-8, while TFM-C increased IL-6 mRNA between 6 and 12 h. To verify whether TFM-C induced an ER stress response in U937 cells, we measured mRNA of HERP and IL-23p19, both of which have been associated with induction of ER stress [24,26,27]. This showed significant up-regulation of both genes by TFM-C while the housekeeping gene GAPDH was not affected (Figure 1D). Viability of U937 cells following exposure to TFM-C was assessed using two different methods (Figure 1B), and showed a limited percentage of apoptotic cells not exceeding 15 to 20% following 12 to 24 h of treatment. Thus, TFM-C blocks cytokine secretion in natural producer cells by ER stress-related mechanisms that may involve repressive effects on both cytokine mRNA production as well as on post-transcriptional and -translational events involved in cytokine secretion, such as the ER-retention coupled to HERP-mediated degradation identified before for IL-12 [23,24,26]. However, of the TFM-C-sensitive cytokines identified in this experiment, IL-1β follows an unconventional protein secretion route involving exocytosis of endolysosome-related vesicles not derived from the ER/Golgi system [28]. Given its blockage by TFM-C, which can not be explained by partial suppression of mRNA levels only, this indicates that TFM-C may suppress secretion of cytokines via interfering with both conventional ER-dependent and unconventional ER-independent transit routes.

### TFM-C inhibits CIA

**Figure 2:**
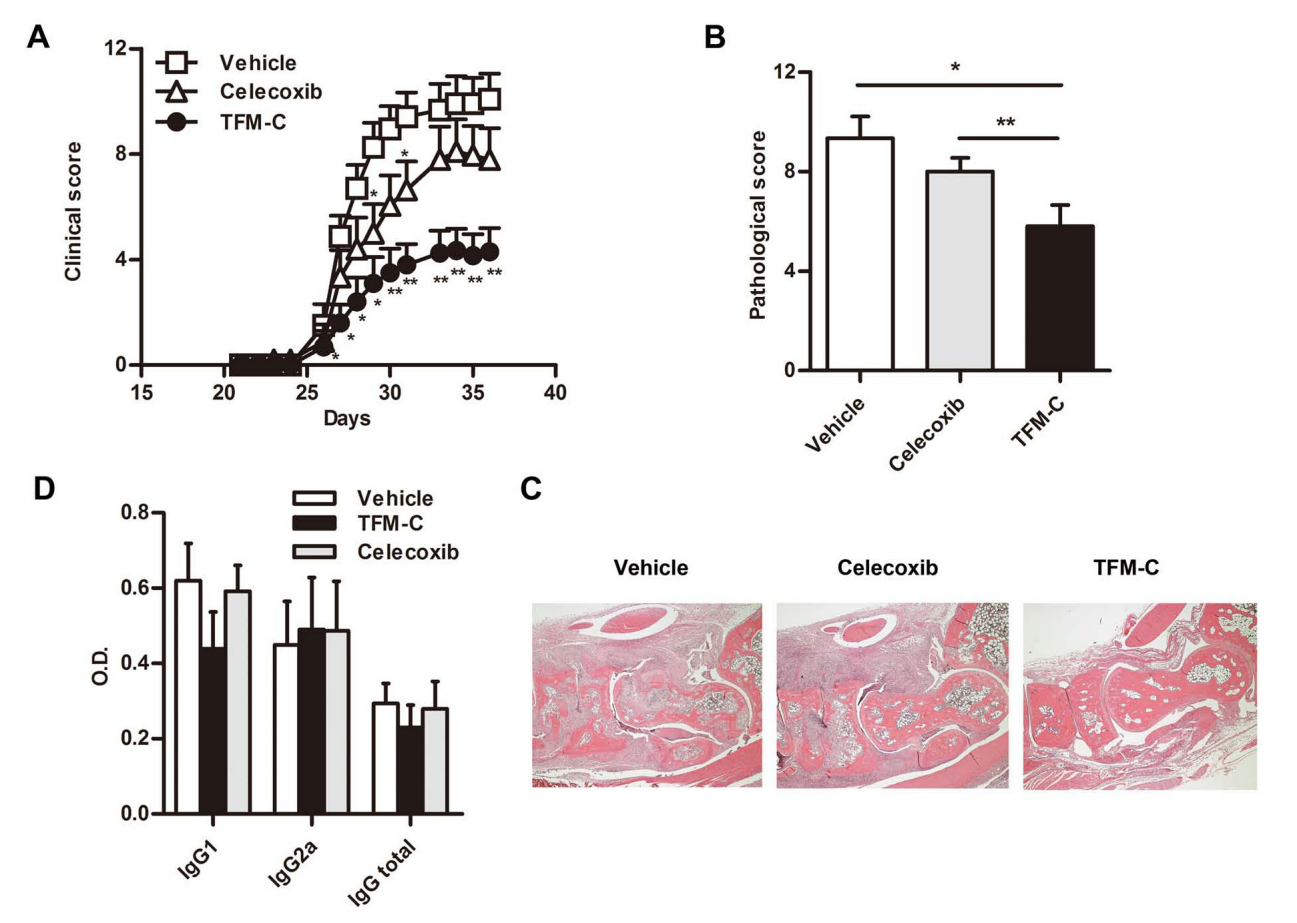
**The effect of TFM-C on CIA**. **A**. Clinical scores of CIA in DBA1/J mice treated with 10 μg/g TFM-C (closed circles), celecoxib (open triangles) or vehicle (open squares) every other day from 21 days after immunization. The data shown are pooled from two similar experiments. Error bars represent + SEM of 10 to 12 mice per group. * *P *< 0.05 compared with control group. * *P *< 0.05 compared with both control and celecoxib-treated groups. **B**. Quantification of histological assessment of joints 37 days after induction of CIA. Result shown is the mean + SEM of five mice per group. * *P *< 0.05, TFM-C-treated versus vehicle-treated group. * *P *< 0.05, celecoxib-treated versus TFM-C-treated group. **C**. Representative histological feature of joints in vehicle-treated (left), TFM-C-treated (right) and celecoxib-treated (middle) mice. (H&E stained; original magnification × 40). **D**. The effect of TFM-C on CII-specific response. CII-specific antibody responses in vehicle- (open bars), TFM-C- (filled bars) and celecoxib-treated (gray bars) group. Individual serum samples were obtained at Day 37 after the induction of arthritis and were analyzed as indicated in Materials and Methods. Data represent the mean + SEM of five mice per group.

First, we examined the effect of TFM-C on CIA induced by immunizing DBA1/J mice with type II collagen. As shown in Figure 2A, administration of TFM-C strongly suppressed the severity of arthritis compared with vehicle-treated mice (*P-*value, < 0.05 by Mann-Whitney *U*-test compared with control from Day 26 and Day 36.). In contrast, administration of celecoxib showed only a mild suppressive effect on CIA, which is consistent with a previous report [19] (*P-*value, < 0.05 by Mann-Whitney *U*-test compared with control at Day 29 and Day 31.) In addition to visual scoring, we analyzed the histological features in the joints of four paws from TFM-C-, celecoxib- or vehicle-treated mice 37 days after disease induction. Quantification of the histological severity of arthritis is shown in Figure 2B and typical histological features are demonstrated in Figure 2C. Arthritis was not apparent in joints treated with TFM-C (Figure 2C, rightmost panel) compared to the severe arthritis with massive cell infiltration, cartilage erosion and bone destruction seen in joints of animals treated with vehicle (Figure 2C, leftmost panel). Both the clinical scores and pathological features of arthritis were significantly less severe in TFM-C-treated mice (Figure 2A-C). The pathological features, including cell infiltration and destruction of cartilage and bone, were slightly less severe in celecoxib-treated mice even though there is no statistically significant difference compared to vehicle-treated mice (Figure 2B). We next examined anti-CII antibody in TFM-C-, celecoxib- or vehicle-treated arthritic mice. There was a trend for reduction in both IgG1 and IgG2a isotypes as well as total IgG anti-CII in TFM-C-treated mice compared to vehicle-treated mice (Figure 2D), but the difference did not reach statistical significance. These results indicate that TFM-C possesses a potent inhibitory effect on CIA compared to vehicle or celecoxib. However, TFM-C treatment had little effect on CII-specific responses.

### TFM-C inhibits CAIA

**Figure 3:**
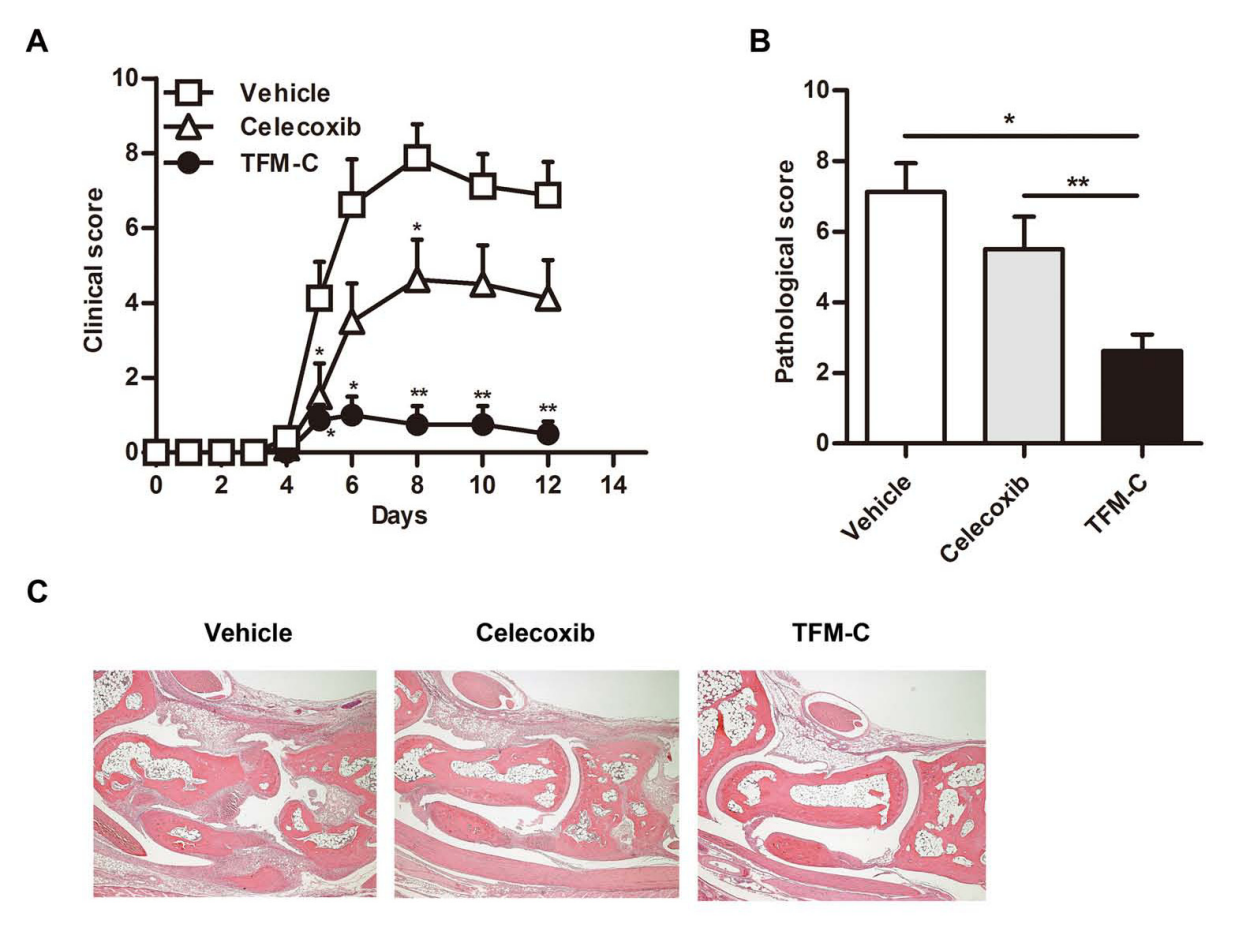
**The effect of TFM-C on CAIA**. **A**. Clinical scores of CAIA in B6 mice treated with 10 μg/g TFM-C (closed circles), celecoxib (open triangles) or vehicle (open squares) every other day from two days before CAIA induction. * *P *< 0.05 compared with control group, * *P *< 0.05 compared with both control and celecoxib-treated groups. Results shown are the mean + SEM of five mice per group. The data shown are from a single experiment representative of two similar experiments. **B**. Quantification of histological assessment of joints 12 days after induction of AIA shown in A. Results shown are the mean + SEM of five mice per group. * *P *< 0.05 control versus TFM-C group, * *P *< 0.05 celecoxib versus TFM-C -treated group. **C**. Representative histological feature of joints in vehicle-treated (left), TFM-C-treated (middle) and celecoxib-treated (right) mice. (H&E stained; original magnification × 40).

Although TFM-C treatment suppressed clinical and pathological severities of CIA, CII-specific antibody levels were not reduced by TFM-C treatment. Therefore, we hypothesized that TFM-C treatment may have a strong inhibitory effect on the effector phase of arthritis. To test this hypothesis, we examined the effect of TFM-C on CAIA induced by injecting a mixture of monoclonal antibodies against type II collagen (CII) followed by lipopolysaccharide (LPS) administration two days later. The major players in CAIA are innate immune cells while adaptive immune cells are not required for disease development. Therefore, CAIA has value as an animal model to study the effector phase of arthritis. In vehicle-treated mice, severe arthritis occurred one week after CII antibody injection, and administration of celecoxib inhibited arthritis slightly (Figure 3A). In contrast, administration of TFM-C significantly suppressed CAIA compared to vehicle or celecoxib treatment. We next analyzed the histological features in the joints of four paws from vehicle-, TFM-C- and celecoxib-treated mice 12 days after disease induction. Quantification of the histological severity of arthritis is shown in Figure 3B and typical histological features are presented in Figure 3C. Massive cell infiltration, cartilage erosion, and bone destruction were observed in joints of vehicle-treated or celecoxib-treated mice but not in those of TFM-C-treated mice (Figure 3B, C). These results indicate that TFM-C exhibits a strong disease inhibitory effect in CAIA in contrast to vehicle or celecoxib.

### TFM-C inhibits the mast cell activation in CAIA

**Figure 4:**
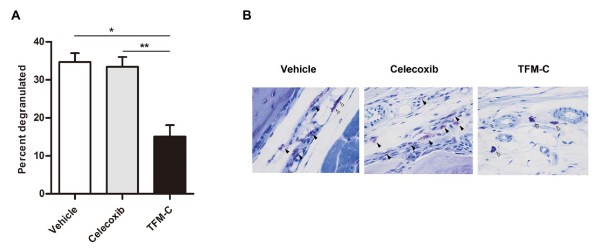
**TFM-C inhibits the mast cell activation in CAIA**. CAIA was induced in B6 mice and the mice were then treated with 10 μg/g TFM-C, celecoxib or vehicle as described in Figure 2. **A**. Quantification of degranulated mast cells in synovium of joints 12 days after induction of CAIA. * *P *< 0.05, compared with vehicle-treated group. * *P *< 0.05, compared with celecoxib-treated group. Results shown are the mean + SEM of six mice per group and were pooled from two experiments. **B**. Hisopathologic features of degranulated or intact mast cells in joints of representative vehicle-, celecoxib- and TFM-C- treated mice (toluidine blue stained; original magnification, × 100). White arrows indicate intact mast cells and black arrows indicate degranulated mast cells.

Next, we sought to understand the mechanism through which TFM-C treatment suppressed arthritis in CAIA. Since mast cells have been demonstrated to be critical for initiation of antibody-induced arthritis [29], we evaluated the effect of TFM-C on the activation of mast cells. Because degranulation is the clearest histological hallmark of mast cell activation, joint mast cells were visually assessed for an intact versus degranulating phenotype after staining with toluidine blue. The proportion of degranulated mast cells was significantly lower in TFM-C-treated mice compared to that in celecoxib- or vehicle-treated mice (Figure 4A, B).

### TFM-C supresses the activation of macrophages

**Figure 5:**
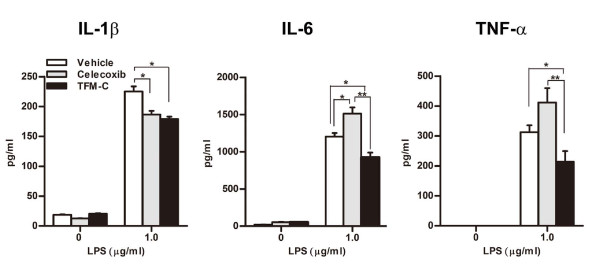
**TFM-C supresses the activation of macrophages**. B6 mice recieved 10 μg/g TFM-C, celecoxib or vehicle on Day 0 and Day 2, and on Day 3, splenic macrophages were collected and were stimulated by LPS *in vitro *in the presence of TFM-C, celecoxib or vehicle. Cytokines were detected by ELISA. IL-1β and IL-6 were measured 24 h after stimulation. TNF-α was measured six hours after stimulation. The data shown are from a single experiment representative of three similar experiments. * *P *< 0.05 compared with control group, * *P *< 0.05 compared with celecoxib-treated group.

Innate immune cells and inflammatory cytokines, such as IL-1 and TNF-α are critical for disease development in CAIA [30]. Thus, we next determined the effect of TFM-C on the production of inflammatory cytokines from macrophages. Splenic macrophages from mice treated with TFM-C, celecoxib or control vehicle, were stimulated with LPS *ex vivo*, and the cytokines in the culture supernatants were measured by ELISA. The production of IL-1, IL-6 and TNF-α from macrophages was efficiently suppressed in TFM-C-treated mice compared to vehicle-treated mice (Figure 5). In celecoxib-treated mice, although the production of IL-1β was decreased, the production of other cytokines such as IL-6 and TNF-α was not suppressed, and the IL-6 production was even enhanced compared to vehicle-treated mice.

### TFM-C suppresses leukocyte influx in thioglycollate-induced peritonitis

**Figure 6:**
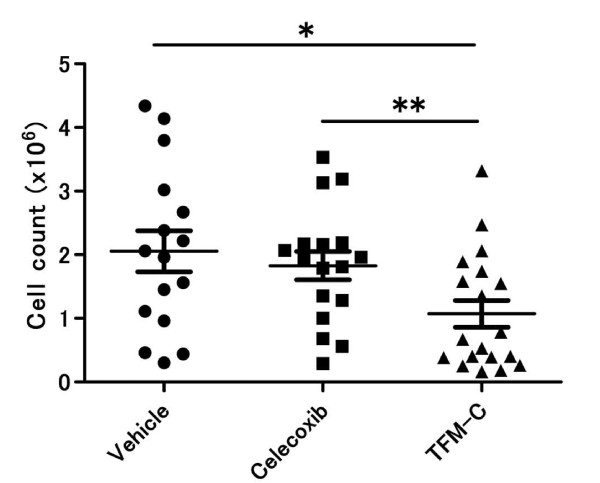
**TFM-C supresses leukocyte influx in thioglycollate-induced peritonitis**. B6 mice recieved 10 μg/g TFM-C, celecoxib or vehicle at two days and one hour before peritoneal injection of thioglycollate. At four hours after thioglycollate injection, peritoneal lavage fluid was collected and the infiltrating cells were counted. Cell numbers are shown from three separate experiments. * *P *< 0.05, TFM-C-treated versus vehicle-treated group. * *P *< 0.05, celecoxib-treated versus TFM-C-treated group.

The other key players in antibody-induced arthritis are neutrophils [31-34]. Neutrophils are recruited to joint tissue and depletion of neutrophils has been shown to supress disease susceptibility and severity in CAIA [35]. An intraperitoneal injection of thioglycollate causes leukocytes influx into the peritoneum from bone marrow and circulation, and neutrophils are the major cell population which first emigrate to the peritoneal cavity. To assess the effect of TFM-C on neutrophil recruitment, mice were treated with TFM-C, celecoxib or control vehicle, and thioglycollate was injected intraperitoneally. Leukocyte cell numbers in the peritoneal cavity four hours after thioglycollate injection were comparable between control and celecoxib-treated groups (Figure 6). However, the peritoneal infiltrating cell numbers were reduced in mice treated with TFM-C, suggesting the suppressive effect of TFM-C on neutrophil recruitment.

Taken together, these results indicate that the activation of innate immune cells, including mast cells, macrophages, and neutrophils, is suppressed in TFM-C-treated mice but not in celecoxib-treated mice.

## Discussion

In the present study we demonstrate, using arthritis models, that TFM-C, a celecoxib analogue with 205-fold lower COX-2-inhibitory activity, inhibits autoimmune disease. TFM-C differs from celecoxib by the substitution of the 4-methyl group by a trifluoromethyl group. This substitution drastically increases the IC_50_s for inhibition of COX1 (15 μM to >100 μM for celecoxib and TFM-C, respectively) and COX2 (0.04 μM to 8.2 μM, respectively), but does not affect the apoptotic index measured in PC3 prostate cancer cells, indicating independence between structural requirements for COX-2 inhibition and apoptosis induction [36]. Celecoxib perturbs intracellular calcium by blocking ER Ca^2+ ^ATPases, and this activity is shared with TFM-C [23,37]. In a HEK293 recombinant cell system, this Ca^2+ ^perturbation is associated with inhibition of secretion and altered intracellular interaction of IL-12 polypeptide chains with the ER chaperones calreticulin and ERp44, and results in the interception of IL-12 by HERP followed by degradation of the cytokine [23,24,26]. While IC_50_s for inhibition of IL-12 secretion by celecoxib or TFM-C are similar [23,24], in the present paper, we show that TFM-C inhibits production of various cytokines from activated macrophages (Figures 1 and 5) and exerts a strikingly stronger inhibitory effect on arthritis models compared to celecoxib. Given that the main biological difference between celecoxib and TFM-C resides in the extent of COX-1 and -2 inhibition, it is, therefore, likely that the less potent effect of TFM-C on COX1/2 inactivation is a contributing, disease-limiting rather than disease-promoting factor in these arthritis models. Indications supporting this concept come from a study showing increased LPS-induced macrophage production of TNF-α by inactivation of COX-2 with celecoxib [38]. Up-regulation of TNF-α by celecoxib was also reported in human PBMCs, rheumatoid synovial cultures and whole blood [20]. The (please delete inverse)relation (please replace correlation with relation)between the anticipated extent of COX inhibition and production of TNF-α was observed in the present study (Figure 5), where activated macrophages showed a tendency toward increased or decreased TNF-α production in the presence of celecoxib or TFM-C, respectively, compared to vehicle-treated cells. In this cell system (Figure 5), celecoxib significantly increased production of the pro-inflammatory cytokine IL-6 while TFM-C suppressed it. Pending future mechanistic studies, this data indicate that prostaglandin-mediated suppressive effects, or other, as yet to be identified differential TFM-C/celecoxib-related effects on TNF-α production may extend to other cytokines as well, and provide an important clue as to the more potent beneficial effects of TFM-C compared to celecoxib in the arthritis models presented here.

The suppression of antibody-induced arthritis, which requires innate but not acquired immune cells [29-34,39], suggests that TFM-C also inhibits the activation of innate immune cells while celecoxib does not. In fact, TFM-C suppresses the production of inflammatory cytokines from macrophages and the activation of mast cells as well as the subsequent recruitment of leukocytes. Mast cells are essential for the initiation of antibody-induced arthritis [29]. Moreover, mast cells are present in human synovia [40-43] and are an important source of both proteases and inflammatory cytokines, including IL-17, in patients with rheumatoid arthritis [42-44]. The clear difference between the effects of TFM-C and celecoxib on the suppression of mast cell activation could explain the differential impact of these compounds on arthritis models. Mast cells are important not only in arthritis but also in other conditions, such as allergy, obesity and diabetes [45]. Therefore, the suppression of mast cell activation by TFM-C may be applicable for the inhibition of these diseases in addition to autoimmune diseases.

Cytokines and chemokines, such as TNF-α and MCP-1, produced by macrophages, are suggested to play important roles for neutrophil influx in thioglycollate-induced peritonitis [46]. Mast cells were shown to produce TNF-α, which recruits neutrophils into the peritoneum in an immune complex peritonitis model [47]. Thus, it is likely that TFM-C suppressed macrophages and mast cells produce such chemoattractants, which in turn inhibited neutrophil influx into the peritoneum. However, it is also possible that TFM-C directly suppressed neutrophil activation. Further studies are required to address this possibility.

As described above, the major players in CAIA are innate immune cells, while adaptive immune cells are not required for disease development. Therefore, CAIA has value as an animal model for the study of the effector phase of arthritis. However, it is well known that adaptive immune cells play a significant role in the pathogenesis of RA and the strongest genetic link in RA is the association with HLA-DR, which is thought to present autoantigens to T cells. The activation of T cells and B cells is believed to initiate and/or enhance the effector inflammation phase of arthritis. In fact, massive infiltration of T and B cells is observed in RA synovium. Therefore, the ideal therapeutic agents for RA are those displaying the capacity to suppress both the induction and effector phases of arthritis. TFM-C treatment suppresses CIA, which requires both innate and adaptive immune cells for the development of arthritis. We previously demonstrated that celecoxib treatment suppresses EAE induced by immunizing B6 mice with myelin oligodendrocyte glycoprotein_35-55 _(MOG) peptide [22]. The suppression of EAE by celecoxib was COX-2 independent and was accompanied by reduced IFN-γ production by MOG-reactive T cells. We observed a trend of reduced anti-CII antibody levels in serum upon TFM-C treatment. As TFM-C inhibited secretion of both recombinant IL-12 and IL-23 using a pIND ponasterone-inducible vector system in HEK293 cells [23,24], TFM-C treatment may have also influenced CII-specific immune responses by suppressing antigen-presenting cells.

Specific inhibition of COX-2 has some adverse effects. Rofecoxib, a highly specific COX-2 inhibitor, was withdrawn from the world market because of an increased rate of cardiovascular events in patients with colorectal polyps [48]. Celecoxib was also shown to augment cardiovascular and thrombotic risk in colorectal adenoma patients, especially in the subgroup suffering from pre-existing atherosclerotic heart disease [49]. Moreover, inhibition of COX-2 activity has been reported to exacerbate brain inflammation by increasing glial cell activation [50]. It has been suggested that the inhibition of COX-2-dependent prostaglandin I_2 _from endothelial cells may be the major cause of thrombosis [51]. As the COX-2-inhibitory activity of TFM-C is 205-fold lower than that of celecoxib, the arthritis suppression by TFM-C appears to be independent of COX-2 inhibition. Therefore, TFM-C, which has strong immunoregulatory abilities but low COX-2-inhibitory activity, could serve as a new disease-modifying agent to prevent the progression of autoimmune diseases such as RA.

## Conclusions

In summary, TFM-C, a trifluoromethyl analogue of celecoxib, inhibits arthritis despite the fact that TFM-C possesses very low COX-2-inhibitory activity. The most striking features of TFM-C are its inhibitory effect on the activation of innate immune cells and its suppression of arthritis compared to celecoxib. TFM-C treatment suppressed both CIA and CAIA by targeting innate immune cells, which are involved in both the induction and the effector phases of arthritis inflammation. Taking these data together, TFM-C may serve as an effective therapeutic drug for arthritis, including RA.

## Abbreviations

B6: C57BL/6J; CII: anti-type II collagen; CAIA: type II collagen antibody-induced arthritis; CFA: complete Freund's adjuvant; CIA: collagen-induced arthritis; COX-2: cycolooxygenase-2; EAE: experimental autoimmune encephalomyelitis; ELISA: enzyme-linked immunosorbent assay; IFA: incomplete Freund's adjuvant; IL: interleukin; LPS: lipopolysaccharide; MOG: myelin oligodendrocyte glycoprotein; *Mtb*: *Mycobacterium tuberculosis*; PBS: phosphate-buffered saline; TFM-C: a trifluoromethyl analogue of celecoxib; TNF: tumor necrosis factor.

## Competing interests

The authors declare that they have no competing interests.

## Authors' contributions

AC, MM, CT, RT and AP performed and evaluated experiments. AC, TY, KV and SM designed and supervised the experiments. IA and KV provided TFM-C. AC, KV and SM prepared the manuscript. All authors have read and approved the manuscript for publication.
